# Peptidoglycan-induced modulation of metabolic and inflammatory responses

**DOI:** 10.1097/IN9.0000000000000024

**Published:** 2023-04-28

**Authors:** Andrea J. Wolf

**Affiliations:** 1 The Karsh Division of Gastroenterology and Hepatology, F. Widjaja Foundation Inflammatory Bowel Disease Institute, Cedars-Sinai Medical Center, Los Angeles, CA, USA; 2 Research Division of Immunology, Department of Biomedical Sciences, Cedars-Sinai Medical Center, Los Angeles, CA, USA

**Keywords:** peptidoglycan, MDP, NOD1, NOD2, metabolism, inflammation

## Abstract

Bacterial cell wall peptidoglycan is composed of innate immune ligands and, due to its important structural role, also regulates access to many other innate immune ligands contained within the bacteria. There is a growing body of literature demonstrating how innate immune recognition impacts the metabolic functions of immune cells and how metabolic changes are not only important to inflammatory responses but are often essential. Peptidoglycan is primarily sensed in the context of the whole bacteria during lysosomal degradation; consequently, the innate immune receptors for peptidoglycan are primarily intracellular cytosolic innate immune sensors. However, during bacterial growth, peptidoglycan fragments are shed and can be found in the bloodstream of humans and mice, not only during infection but also derived from the abundant bacterial component of the gut microbiota. These peptidoglycan fragments influence cells throughout the body and are important for regulating inflammation and whole-body metabolic function. Therefore, it is important to understand how peptidoglycan-induced signals in innate immune cells and cells throughout the body interact to regulate how the body responds to both pathogenic and nonpathogenic bacteria. This mini-review will highlight key research regarding how cellular metabolism shifts in response to peptidoglycan and how systemic peptidoglycan sensing impacts whole-body metabolic function.

## 1. Introduction

**Figure 1.**
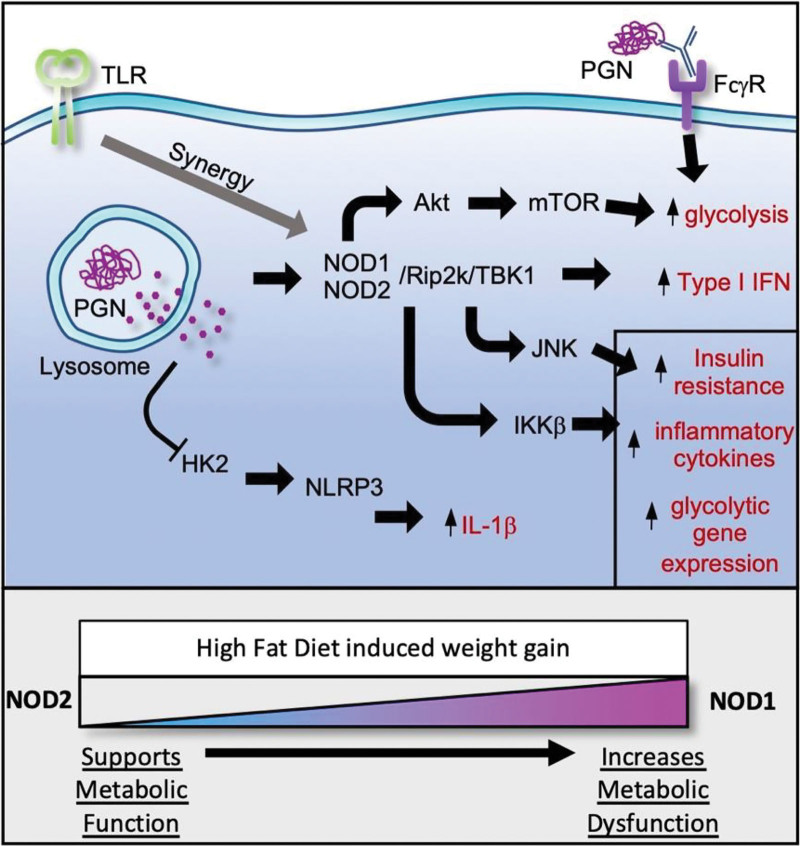
**Peptidoglycan and its fragments activate several signaling pathways that impact the inflammatory and metabolic response of the cells.** This is an overview of the main pathways that are activated by peptidoglycan in macrophages and some of the inflammatory outcomes. There is a lot of crosstalk between the pathways and in the context of the whole bacteria where TLR signaling can synergize with peptidoglycan by many of the same pathways, the outcome is extremely context-dependent. But these highlight the responses that can be linked to peptidoglycan specifically. The bottom panel illustrates the current counterbalancing roles that NOD1 and NOD2 play in balancing whole-body metabolism during diet-induced obesity. NOD1 and 2, nucleotide-binding oligomerization domain 1 and 2; TLR, toll-like receptor.

Crosstalk between the innate immune system and the metabolic systems of both immune and nonimmune cells is an expanding area of research. Even during the early years of innate immune research, it was clear that in response to inflammatory stimuli, myeloid cells shifted their metabolism to rely predominantly on glycolysis for energy production ^[1]^. More recently, there has been a growing body of research exploring the intricate and essential shifts in cellular metabolism that accompany and shape the innate immune responses to microbes. In addition, there is a growing appreciation that microbial ligands derived from the gut microbiota impact whole-body metabolic regulation contributing to metabolic diseases. This mini-review will focus on how recognition of bacterial cell wall peptidoglycan fragments impacts not only cellular but also whole-body metabolic systems and associated inflammation (**Figure 1**).

Peptidoglycan is a polymeric structure composed of a sugar backbone of repeating subunits of *n*-acetylglucosamine and *n*-acetylmuramic acid that are crosslinked by amino acid sidechains ^[2–4]^. Peptidoglycan is the dominant component of the cell wall of Gram-positive bacteria but is also a component of the cell wall of Gram-negative bacteria, though less abundant, making it an important innate immune ligand in the body’s recognition of all bacteria. Peptidoglycan degradation products themselves are innate immune ligands ^[5–7]^, but peptidoglycan, based on its degradation sensitivity, also regulates access to additional innate immune ligands making it a vital factor influencing innate immune recognition of bacteria ^[8,9]^. A comparison of wild-type *Staphylococcus aureus,* with degradation-insensitive peptidoglycan, and a degradation-sensitive mutant found a significant elevation in the quantity and duration of the tumor necrosis factor (TNFα), interleukin-6 (IL-6), and nitric oxide (NO) produced by macrophages when the bacteria’s peptidoglycan is degradation sensitive ^[8]^. This highlights the inflammatory and structural importance of peptidoglycan. However, peptidoglycan’s sugar backbone and amino acid sidechain composition varies significantly between different strains of bacteria and often undergo significant modifications ^[2,10]^, consequently, many of the observed impacts of peptidoglycan on the innate immune system and the metabolic system may not always be broadly applicable. Direct examination of the inflammatory and metabolic differences driven by peptidoglycan modifications in different bacterial strains is a significantly understudied area of research.

Peptidoglycan fragments are recognized by both immune and nonimmune cells throughout the body by numerous receptors. The first identified peptidoglycan receptors were the nucleotide-binding oligomerization domain 1 and 2 (NOD1 and NOD2) ^[11–13]^. NOD1 and NOD2 are cytosolic receptors that directly detect fragments of peptidoglycan shed from bacteria or generated during the degradation of bacteria in the phagolysosome. NOD1 primarily detects peptidoglycan motifs found in Gram-negative bacteria ^[11,13]^, whereas NOD2 primarily detects peptidoglycan motifs found in Gram-positive bacteria ^[14]^. Experimentally, synthetic fragments of peptidoglycan specific for NOD1 such as γ-d-glutamyl-meso-diaminopimelic acid (iE-DAP) and l-Ala-γ-d-Glu-meso-diaminopimelic acid (Tri-DAP), or specific for NOD2, such as muramyl dipeptide (MDP), are the minimal motifs able to activate receptors for peptidoglycan and are often used to mimic peptidoglycan ^[6,7]^. Recent work by Stafford et al ^[15]^ showed that the metabolite recycling enzyme, *n*-acetylglucosamine kinase (NagK), is necessary for NOD2 sensing of synthetic peptidoglycan ligand MDP. The authors showed that both MDP and peptidoglycan fragments need to be phosphorylated by NagK to be recognized by NOD2. Further work is necessary to determine the importance of NagK to the recognition of whole bacteria in vivo. However, this is an interesting example of metabolic pathways playing a role in sensing peptidoglycan.

Another place where metabolic pathways play a role in peptidoglycan sensing is in the activation of the inflammasome by peptidoglycan. The inflammasome is a multiprotein signaling complex also found in the cytosol that regulates the activation of the proteolytic enzyme caspase-1 which plays a vital role in controlling the production of potent inflammatory cytokines such as IL-1β and IL-18 ^[16]^. Degradation of peptidoglycan in the macrophage lysosome produces fragments that trigger activation of the NOD-like receptor family pyrin domain containing 3 (NLRP3) inflammasome ^[17]^. Subsequent research found that the *n*-acetylglucosamine subunit of the peptidoglycan sugar backbone was sufficient to activate the NLRP3 inflammasome leading to IL-1β production by macrophages ^[5]^. This activation was linked to *n*-acetylglucosamine inhibition of the glycolytic enzyme hexokinase ^[5]^ illustrating an additional level of crosstalk between cellular metabolism and innate immune sensing. The synthetic peptidoglycan ligand MDP actives the NOD-like receptor family pyrin domain containing 1 (NLRP1) and NLPR3 inflammasome ^[18,19]^ indicating that multiple peptidoglycan ligands may collaborate to induce inflammasome activation.

Often peptidoglycan is listed as a ligand for toll-like receptor 2 (TLR2), however, this is a controversial topic. Due to the complex crosslinked polymeric structure of peptidoglycan, several studies have concluded that commercial peptidoglycan preparations have significant lipoprotein contamination leading to strong TLR2 stimulating capacity ^[20–22]^. Meanwhile, other studies have modeled TLR2 binding to possible peptidoglycan fragments ^[23]^, have measured the binding of peptidoglycan fragments to TLR2 by surface plasmon resonance ^[24]^, or used bacterial mutants to try to eliminate lipoproteins from peptidoglycan preparations and still found some level of TLR2 activity in reporter cells ^[25]^. The physiological importance of possible peptidoglycan binding to TLR2 is unclear given that peptidoglycan is often encountered in the presence of other stronger TLR2 agonists. What is clear is that peptidoglycan synergizes with other TLR ligands from the bacteria to influence the overall inflammatory response. To focus this mini-review on the metabolic impacts of peptidoglycan, this introduction is necessarily limited to a brief description of the relevant receptors. For a more in-depth examination of the signaling pathways for each of these innate immune receptors, the reader is encouraged to read one of the many comprehensive reviews that can provide that additional signaling details ^[13,16,26]^.

## 2. Myeloid cell responses to peptidoglycan

### 2.1 Peptidoglycan-induced changes in glycolysis and mitochondria function

Peptidoglycan is primarily recognized by phagocytic cells of the immune system, which include macrophages, dendritic cells, and neutrophils. Peptidoglycan’s polymeric structure requires phagocytosis and enzymatic degradation in lysosomes to liberate the ligands contained within the structure, and phagocytic cells are uniquely equipped for this process. Most of the research characterizing peptidoglycan signaling pathways and metabolic changes has been performed in primary macrophages and macrophage cell lines. Stimulation of myeloid cells with peptidoglycan, iE-DAP, or MDP leads to an increase in glycolytic gene expression and an acute transient increase in glycolysis ^[27,28]^. However, a comprehensive understanding of how NOD and TLR receptors synergize and regulate glycolysis in response to particulate peptidoglycan has yet to be undertaken. Nonetheless enhanced glycolysis seen in response to synthetic peptidoglycan ligands is the same response observed during classic activation of TLR4 in macrophages by lipopolysaccharide (LPS), which is used extensively to model the metabolic shifts in macrophages in response to proinflammatory stimuli ^[1]^. Like LPS, the NOD1 and NOD2 ligands, Tri-DAP and MDP, lead to an acute increase in glycolysis during the first 2 hours as measured by extracellular acidification (ECAR) in human monocyte macrophages, but this glycolytic change is absent by 24 hours ^[27]^. Using the glycolytic inhibitor, 2-deoxyglucose (2-DG), research has generally shown that glycolysis is necessary for at least some inflammatory cytokine production in myeloid cells in response to numerous innate inflammatory ligands ^[29–32]^ though the opposite effect has also been observed ^[33]^. In human monocytes, 2-DG inhibition of glycolysis during stimulation with the NOD1 ligand Tri-DAP did not impact cytokine production under normoxic conditions but did impair cytokine production under hypoxic conditions ^[27]^. However, 2-DG treatment was also found to induce endoplasmic reticulum (ER) stress and an unfolded protein response, which affected responses independent of an impact on glucose utilization ^[27]^. Due to the nonspecific impacts of 2-DG, it is not an ideal tool to examine the importance of increased glycolysis during peptidoglycan stimulation. As an alternative approach for assessing the importance of glycolysis during inflammation, macrophages deficient for the glucose transporter 1 (Slc2a1), which is important for supporting glycolysis, showed reduced inflammatory responses to LPS ^[34]^. Unfortunately, LPS was the only innate immune ligand tested in these experiments, so further work will need to be carried out using similar genetic models to fully untangle the importance of glycolytic changes to peptidoglycan-induced inflammatory responses.

Treatment of macrophages with whole particulate peptidoglycan or soluble NOD1 or NOD2 ligands (iE-DAP or MDP, respectively) leads to an increase in protein kinase B (a.k.a Akt) and mammalian target of rapamycin (mTOR) activation, both of which are known to modulate metabolic systems in cells ^[27,35,36]^. Akt phosphorylation results in increased mitochondrial localization of the glycolytic enzyme hexokinase 2 ^[27]^, which is believed to enhance the glycolytic rate of cells ^[30]^. Treating macrophages with an Akt inhibitor blocks NOD1-induced acute increases in ECAR, a measure of lactate production and cellular glycolysis, however, inhibition of Akt only affects NOD1-induced cytokine responses, not LPS responses ^[27]^. Though there are some common metabolic changes in response to LPS and peptidoglycan ligands, the impact on inflammation may vary based on the receptor/s that are activated.

Although increased glycolysis does seem to be a common response to peptidoglycan ligands, when peripheral blood mononuclear cells from NOD2 deficient humans were stimulated with mycobacteria there was a decrease in the production of certain cytokines but no decrease in lactate production ^[28]^ suggesting maybe peptidoglycan signaling through NOD2 does not impact glycolysis as much as other studies suggest. Mouse bone-marrow macrophages from NOD1 and NOD2 single- or double-knockout mice also show altered cytokines production in response to mycobacteria but no reduction in lactate production ^[28]^. But lactate production from mycobacteria-treated TLR2^−/−^ cells was reduced, indicating an essential role for TLR2 in glycolytic regulation but not NOD sensing in response to whole mycobacteria ^[28]^. Therefore, in the context of whole bacteria, the metabolic changes that occur in macrophages may be indirectly regulated by peptidoglycan based on the degradation sensitivity of the peptidoglycan being studied, which has been shown to control the availability of TLR2 and TLR9 ligands in Gram-positive bacteria ^[8,9]^. Unfortunately, because each bacteria modifies its peptidoglycan uniquely, impacting the exact ligand mixture, the role of peptidoglycan in bacterial sensing and metabolic changes will likely require experimentation using the relevant factors for each unique microbe. Another factor to consider is that in response to LPS, human and mouse macrophages demonstrate divergent changes in metabolism ^[37]^. Thus far a side-by-side comparison of human and mouse cells stimulated with peptidoglycan or synthetic ligands has not been undertaken, though mouse vs human differences could account for some of the variability in the reported metabolic changes induced by peptidoglycan and will have to be considered in future studies.

Although there is a lot of evidence that peptidoglycan increases myeloid cell glycolysis the impact on mitochondrial function is much less clear. In human monocytes, when NOD1 and NOD2 ligands induced an acute increase in glycolysis there was no concurrent change in mitochondrial oxygen consumption rate (OCR) in response to soluble NOD1 or NOD2 ligands ^[27]^. When macrophages were stimulated with MDP together with the TLR2 ligand Pam_3_CSK_4_ they synergized leading to the phosphorylation of ataxin-3 in a NOD2/Rip2k/TBK1-dependent manner (Rip2K-receptor-interacting serine/threonine-protein kinase 2, TBK-1-TANK-binding kinase). Ataxin-3 localizes to mitochondria cristae protein MIC60 and is important for the regulation of oxidative phosphorylation complex I proteins expression ^[38]^. Knocking down ataxin-3 reduces basal mitochondrial oxygen consumption and prevents MDP/Pam3CSK4-induced mitochondrial reactive oxygen species (ROS), impairing the ability of the cells to kill *Salmonella typhimurium* ^[38]^, but whether it was the mitochondrial oxygen consumption, ROS, or both are important for bacterial killing is unclear. Conversely, MDP can also induce NOD2-dependent IL-10 ^[39]^, which has been shown to counter mTOR activation and dampen glycolysis, and NO production ^[40]^, which can inhibit mitochondrial oxygen consumption ^[41–43]^. With all these counterbalancing metabolic signals, the exact shift in mitochondrial function in response to peptidoglycan and its ligands will be dependent on the exact mix of ligands, cytokines, and signaling intermediates that are produced in response to specific bacteria. What happens to glycolysis and oxygen consumption in response to particulate peptidoglycan is less clear and will require study in NOD and TLR2 deficient cells to untangle the crosstalk between the two pathways to fully understand the complete metabolic impact (**Figure 1**). But consistent with other innate immune ligands peptidoglycan signaling seems to generally increase glycolysis but the impact on mitochondrial oxygen consumption seems more mixed.

### 2.2 Peptidoglycan-induced signaling pathways that impact metabolism

The metabolic shifts that occur in response to peptidoglycan are impacted by several signaling pathways. For example, the inhibitor of nuclear factor-kβ kinase (IKKβ) and c-Jun *N*-terminal kinases (JNK) signaling pathways are activated by both TLR and NOD ligands ^[44]^. IKKβ and JNK activation leads to serine phosphorylation of insulin receptor substrate 1 and reduction in insulin sensitivity in monocyte, hepatocyte, and adipocyte ^[28,45–47]^. In macrophages reduced insulin sensitivity shifts the cells toward a less inflammatory or more trained immunity phenotype ^[48,49]^, which may have longer-term impacts on macrophage responsiveness.

Mycobacteria infection also strongly activates the NOD2/Rip2k/interferon-regulatory factor-5 signaling pathway in macrophages resulting in the production of type I interferons, which is reduced in NOD2^−/−^ macrophages supporting the conclusion this is a peptidoglycan-driven response ^[50]^. Type I interferons induced specifically by live mycobacteria dampen the rate of glycolysis, which is normally enhanced by innate immune ligands, and increased the mitochondrial damage in macrophages in an interferon alpha receptor (IFNAR)-dependent manner ^[51]^. Although these studies hint at a role for peptidoglycan-induced type I interferon responses unlike live mycobacteria, live *S. aureus* and *S. aureus* peptidoglycan do not induce type I interferon production from dendritic cells ^[52]^. Therefore, the induction and subsequent metabolic modulation by type I interferons in response to peptidoglycan is likely to vary significantly between bacterial strains as are the type I interferon-induced metabolic changes.

Peptidoglycan also leads to the production of prostaglandin E2 (PGE2) from macrophages starting at 2 hours and peaking at 24 hours. Blocking PGE2 production with cyclooxygenase inhibitor blocks peptidoglycan-induced IL-6 production ^[53]^ and inducible nitrogen oxide synthase (iNOS) expression ^[54,55]^. PGE2 treatment of alternatively activated macrophages reduced mitochondrial function and glycolysis ^[54]^. The impact of PGE2 on mitochondrial function may be a result of increased iNOS expression induced by PGE2 leading to high levels of NO production, which has been shown to inhibit mitochondrial complex IV function ^[56]^. But while PGE2 may be an important peptidoglycan-induced inflammatory response, the importance of PGE2 in peptidoglycan-induced metabolic changes needs more research to be fully understood.

Finally, a hallmark of the LPS-induced metabolic shift in macrophages is increased expression of aconitate decarboxylase 1 (ACODI, IRG1), which shifts citrate from the TCA cycle into the itaconate production pathway ^[57]^. Itaconate is an important antimicrobial metabolite and inhibits succinate dehydrogenase disrupting the TCA cycle and oxidative phosphorylation ^[58,59]^. However, stimulation with the NOD1 ligand, Tri-DAP, only slightly increases ACOD1 compared to LPS ^[27]^ which may explain the lack of an impact on macrophage oxygen consumption (OCR) in response to NOD1 ligands alone ^[27]^, but when NOD signals synergize with TLR signals during bacterial infection this important antimicrobial pathway is likely enhanced.

### 2.3 Additional peptidoglycan recognition pathways

There are a few other pathways that are involved in peptidoglycan recognition that are linked to metabolic changes that are worth mentioning and considering further in the future. First, peptidoglycan degradation also generates *n*-acetylglucosamine which was found to induce NLRP3 inflammasome activation through inhibition of the glycolytic enzyme hexokinase ^[5]^. Hexokinase inhibition leads to its dissociation from mitochondria and triggers NLRP3 activation ^[5]^. This is an interesting observation where a metabolic protein is contributing to the sensing of microbial ligands. It also begs the question do the sugars generated during bacterial degradation directly alter myeloid cellular metabolism? This possibility has yet to be addressed. But the NLRP3 inflammasome activating capacity of peptidoglycan from different strains of bacteria was linked to the differential *n*-acetylglucosamine content of each strain’s peptidoglycan ^[5]^ reinforcing the need for the study of strain-specific peptidoglycan-induced responses.

Second, peptidoglycan recognition proteins (PGRP) are the important receptors for peptidoglycan that impact inflammation and cellular function throughout the body ^[60]^ but so far PGRP’s impact on metabolism has only been examined in limited studies. THP-1 macrophage cell line stimulation with PGRP-peptidoglycan complexes resulted in increased mitochondrial metabolic activity as well measured by resazurin incorporation and enhance inflammatory responses ^[61]^. Future studies focusing on the metabolic impacts of PGRP recognition of circulating peptidoglycan fragments are vital and will likely shed light on additional mechanisms of metabolic regulation.

Third, peptidoglycan can also be opsonized by serum antibodies that are found almost ubiquitously in the human bloodstream ^[62]^. Coggeshall et al demonstrated that opsonization and FcγR engagement are important for recognition and inflammation induced by macrophages in response to *Bacillus anthracis* peptidoglycan ^[62]^. Although metabolic shifts were not directly measured in these studies, FcγR crosslinking leads to increased transcription of glycolytic genes, increased glycolysis, and decrease oxygen consumption in both mouse and human macrophages ^[63,64]^. In a mouse model of lupus-like immune complex-mediated nephritis, the macrophage FcγR-induced glycolytic switch was found to be a good target for modulating the inflammatory responses ^[63]^. Whether circulating peptidoglycan fragments can form immune complexes and induce similar inflammatory and metabolic changes is an underexplored possibility.

## 3. Peptidoglycan-induced changes in cellular metabolism of nonimmune cells

### 3.1 Adipose tissue

Even though peptidoglycan is generally a polymeric structure that is tightly associated with whole bacteria, peptidoglycan fragments are shed from bacteria during cell wall restructuring associated with processes such as bacterial division ^[2]^. In addition, peptidoglycan fragments have been found in the circulation of mice with no obvious infection ^[65,66]^. Germ-free mice have significantly lower levels of circulating NOD1 and NOD2-specific peptidoglycan fragments ^[67]^, indicating that the microbiome is a significant source of circulating peptidoglycan fragments. Under certain conditions, such as high-fat diet feeding, where intestinal permeability is increased, there are elevated levels of peptidoglycan fragments in the circulation ^[68]^. Circulating peptidoglycan fragments can be a significant source of inflammatory signals. For example, systemic administration of peptidoglycan fragments can lead to arthritis, and during a *B. burgdorferi* infection model of Lyme disease, peptidoglycan fragments were detected in circulation ^[69]^. The implication is that cells throughout the body without strong phagocytic function, but that express NOD1 and/or NOD2, are also likely to encounter peptidoglycan ligands. Several nonimmune cell subsets express NOD1 and/or NOD2 such as adipocytes, which express NOD1 ^[70]^. White adipose tissue (WAT) explants stimulated with the NOD1 ligand KF565 leads to increased lipolysis that was not observed in NOD1^−/−^ WAT. The adipocyte cell line 3T3-L1 increases lipolysis in response to peptidoglycan-derived NOD1 ligands ^[70,71]^. However, stimulation with the NOD2 ligand MDP does not increase adipocyte lipolysis ^[71]^, either due to lower NOD2 expression or differential signaling by the two receptors in adipocytes. Another possibility given recent research is that MDP phosphorylation by NagK to generate the proper NOD2 ligand ^[15]^ is impaired in adipocytes leading to diminished NOD2 ligand sensing. The lipolysis induced by KF565 activation of NOD1 is a result of the extracellular signal-regulated kinase and protein kinase A signaling ^[70]^, which should also be activated in response to NOD2 signaling. Unlike in adipocyte cell lines, MDP may impact human primary adipocyte differentiation, so one explanation for the discordant results is differential NOD2 expression in primary cells compared to cell lines ^[71]^. The field would benefit from a more comprehensive examination of NOD1 and 2 expressions and signaling in primary adipocytes to better understand how circulating peptidoglycan fragments may impact adipocytes, especially in models of metabolic stress.

### 3.2 Intestinal epithelium

NOD2 also plays an important role in the maintenance of intestinal epithelium ^[72]^. NOD2 polymorphisms are some of the strongest genetic associations found in Chron’s disease ^[72,73]^. Tonic signaling through NOD2 from microbiota-derived NOD2 ligands leads to increased production of defensins by Paneth cells, which is important for maintaining the proper balance of the gut microbiota ^[74]^. NOD2 signaling also increases mucus production by goblet cells ^[75]^ and counterbalances the TLR4 signals leading to excessive TNF-α production that can damage epithelial cells and increase intestinal permeability ^[76–78]^. This is an example of NOD2 ligand recognition in nonimmune cells serving an essential homeostatic function in the body the impact of this explored further in the next section.

## 4. The impact of peptidoglycan on whole-body metabolic function

### 4.1 Role of NOD1

In healthy volunteers, antibodies generated against MDP can detect stable levels of peptidoglycan fragments in the serum at 0.16–1 mg/mL in 75% of subjects and 4–5 mg/mL in 22% of subjects ^[79]^. Due to the presence of peptidoglycan fragments in the circulation ^[65,66]^, peptidoglycan-induced NOD1 and NOD2-driven changes in whole-body metabolic function are important areas of study. Within 1 week of being on a high-fat diet, there are increased bacteria in adipose tissue, and this is reduced in NOD1^−/−^ mice ^[80]^. NOD1 detection of Gram-negative bacterial ligands is necessary for the shift in glucose and insulin responsiveness in mice, the translocation of bacteria across the intestinal epithelium, and the inflammation associated with the presence of bacteria in adipose tissue ^[46,47,80,81]^. However, the NOD1-expressing cell type that is responsible for each of these outcomes is unclear. One of the key tissues that respond to NOD1 ligands is adipose tissue. Diet but not genetic-induced obesity leads to increased NOD1 expression in adipose tissue ^[46]^. However the fold increase in NOD1 gene expression is more than NOD2, it is unclear if the overall expression of NOD2 is low or just not increased. NOD1 knockdown decreased adipocyte responses to the NOD1 ligand Tri-DAP ^[46]^. NOD1 activation in adipocytes can lead to inflammatory responses and reduced insulin sensing and glucose uptake ^[46]^. Pancreatic islet cells are another metabolically important cell type impacted by peptidoglycan ligands. NOD1 ligands from gut microbiota can stimulate insulin trafficking in islet cells ^[82]^. However, tissue-specific deletion of NOD1 has yet to be undertaken to determine if peptidoglycan sensing in either adipocytes or islet cells is regulating systemic metabolic changes.

### 4.2 Role of NOD2

The importance of NOD2 to whole-body metabolism is illustrated by the fact that high-fat diet-fed NOD2^−/−^ mice exhibit exacerbated insulin resistance, impaired glucose tolerance, and increased adipose tissue inflammation, but there is no difference in overall body mass compared to controls ^[83,84]^. Using bone-marrow chimeric mice, the difference was attributed to a role for NOD2 in nonhematopoietic cells. NOD2^−/−^ mice had more bacteria in adipose tissue following oral gavage of *Escherichia coli*. Bacterial translocation may be due to increased bacterial adherence to the mucosa in NOD2^−/−^ mice and in NOD1^−/−^ mice ^[83]^. Because only the translocation of orally gavaged *E. coli,* not endogenous gut microbes, was measured it is unclear if the increased translocation is specific to *E. coli* strains or a result of the levels of bacteria that were gavaged. A comparison of the translocation of orally gavaged Gram-positive and Gram-negative bacteria in high-fat diet-fed mice would be interesting and would help pars out the importance of different types of bacteria during metabolic disease. NOD2^−/−^ also leads to changes in liver metabolism and inflammation ^[83]^. In addition, NOD2^−/−^ mice may have gut microbiome dysbiosis in both chow and high-fat diet-fed mice. Colonization of germ-free mice with the microbiota from high-fat diet-fed NOD2^−/−^ mice transferred many, but not all metabolic and inflammatory changes to germ-free mice ^[83,84]^. Based on the impaired metabolic function and more severe inflammation in the NOD2^−/−^ mice the conclusion is that NOD2 signaling is protective against metabolic disease ^[83,85]^ and this protection requires Rip2k signaling ^[85]^. In contrast, NOD1 signaling negatively influences metabolic disease. The protective role for NOD2 and recognition of peptidoglycan fragments in metabolic disease is also supported by experiments in a mouse model of malnutrition that found that oral gavage with live *Lactiplantibacillus plantarum* or cell wall isolates enhanced the weight gain and growth of the mice in a NOD2-dependent manner despite the nutrient restriction ^[86]^. Using tissue-specific knockout mice, the impact of *L. plantarum* was determined to be mediated by NOD2 expression in intestinal epithelial cells (IEC), and IEC-specific MyD88^−/−^ mice still showed increased growth and weight gain in response to *L. plantarum* ruling out a role of TLR signaling ^[86]^. Although the exact link between NOD2 signaling in IEC and weight gain are not fully elucidated, the study found an increase in type I interferon production and increased responsiveness of the liver to growth hormone leading to increased insulin-like growth factor 1 production ^[86]^. Weight gain is not only impacted by NOD2 signaling in the intestine but studies looking at a lack of NOD2 expression in inhibitory γ-aminobutyric acid transporter–positive (GABAergic) neurons found increased weight gain and poor temperature regulation in older female mice ^[87]^. These are clear examples that the expression of NOD2 and recognition of microbiota-derived peptidoglycan fragments in circulation impact numerous cell types throughout the body to positively impact the overall balance of the body’s metabolic systems. However, when NOD1/2 double-knockout mice were studied they were protected from diet-induced metabolic disease including inflammation and insulin resistance, and showed lower lipid accumulation in WAT ^[88]^. This suggests that on balance the impact of NOD1 activation is a stronger influence on metabolic disease than the protective signals from NOD2.

In addition to NOD1 and NOD2, NLRP3 activation is linked to more severe metabolic disease ^[89]^ and bacterial peptidoglycan is a known activator of NLRP3 ^[5,17]^, but experiments directly testing the impact of peptidoglycan on NLRP3-induced metabolic changes have not been reported and will require further study.

## 5. Conclusions

All of the research highlighted here paints a picture of the importance of peptidoglycan during infection and homoeostatic interactions with the gut microbiota. Peptidoglycan strongly influences cellular and whole-body metabolism and inflammation (**Figure 1**). Difficulties can arise in assembling a clear picture of all these different signaling pathways activated in response to peptidoglycan and how they impact metabolism because of the diverse reagents utilized throughout the studies. For example, some of the studies use crude peptidoglycan which is particulate and a mixture of NOD and TLR ligands, others only examine the response to soluble synthetic ligands such as MDP, and others combine MDP with synthetic TLR2 ligands. Because peptidoglycan is experienced by the body as both free NOD1 and NOD2 ligands and in the context of the whole microbes where TLR ligands can synergize to enhance responses ^[40,90]^, it is extremely important to use genetic knockouts to understand what pathways are being activated by peptidoglycan in each context and that data is not always available. With the ever-expanding repertoire of genetic tools available for the manipulation of mice and cells hopefully some of these pathways will be reexamined more comprehensively.

Another complication that has recently become apparent is that many of the studies use NOD1 and NOD2 activation in vivo as a surrogate for peptidoglycan sensing, but NOD1 and NOD2 can also be activated by certain lipids ^[47,81]^ and there may be endogenous ligands, such as sphingosine-1 phosphate, that can activate NOD1 and NOD2 ^[91]^. Some studies of metabolic dysfunction using NOD1 and NOD2 deficient mice were conducted in germ-free mice, devoid of circulating peptidoglycan fragments, strongly supporting a role for circulating peptidoglycan in metabolic disease. But additional studies will need to be performed to determine if NOD1 and NOD2 nonmicrobial ligand sensing are contributing to associated metabolic dysfunction. As mentioned throughout this review, each strain of bacteria uniquely modifications its peptidoglycan, therefore understanding the peptidoglycan from different bacterial strains with unique modifications impacts the inflammation and metabolic response is an area that will require additional research to fully understand. There is no double that future studies will clarify and solidify the important role that peptidoglycan sensing plays in regulating metabolism in response to both pathogenic and nonpathogenic microbes.

## Conflicts of interest

The author declares that he has no conflicts of interest.

## Funding

This work was supported by grant R01AI148465 “Mechanisms of peptidoglycan-induced modulation of metabolic and inflammatory responses to bacteria”.
